# HSP10 as a Chaperone for Neurodegenerative Amyloid Fibrils

**DOI:** 10.3389/fnins.2022.902600

**Published:** 2022-06-13

**Authors:** Johan N. K. Larsson, Sofie Nyström, Per Hammarström

**Affiliations:** Department of Physics, Chemistry and Biology, Linköping University, Linköping, Sweden

**Keywords:** amyloid, GroES, misfolding, aggregate, proteostasis, HSP10

## Abstract

Neurodegenerative diseases (NDs) are associated with accumulated misfolded proteins (MPs). MPs oligomerize and form multiple forms of amyloid fibril polymorphs that dictate fibril propagation and cellular dysfunction. Protein misfolding processes that impair protein homeostasis are implicated in onset and progression of NDs. A wide variety of molecular chaperones safeguard the cell from MP accumulation. A rather overlooked molecular chaperone is HSP10, known as a co-chaperone for HSP60. Due to the ubiquitous presence in human tissues and protein overabundance compared with HSP60, we studied how HSP10 alone influences fibril formation *in vitro* of Alzheimer’s disease-associated Aβ1–42. At sub-stoichiometric concentrations, eukaryotic HSP10s (human and *Drosophila*) significantly influenced the fibril formation process and the fibril structure of Aβ1–42, more so than the prokaryotic HSP10 GroES. Similar effects were observed for prion disease-associated prion protein HuPrP90–231. Paradoxically, for a chaperone, low concentrations of HSP10 appeared to promote fibril nucleation by shortened lag-phases, which were chaperone and substrate dependent. Higher concentrations of chaperone while still sub-stoichiometric extended the nucleation and/or the elongation phase. We hypothesized that HSP10 by means of its seven mobile loops provides the chaperone with high avidity binding to amyloid fibril ends. The preserved sequence of the edge of the mobile loop GGIM(V)L (29–33 human numbering) normally dock to the HSP60 apical domain. Interestingly, this segment shows sequence similarity to amyloidogenic core segments of Aβ1–42, GGVVI (37–41), and HuPrP90-231 GGYML (126–130) likely allowing efficient competitive binding to fibrillar conformations of these MPs. Our results propose that HSP10 can function as an important molecular chaperone in human proteostasis in NDs.

## Introduction

Protein misfolding and aggregation is associated with a large group of diseases. While the field has generated crucial knowledge and research is expanding, treatments and diagnostic methods for protein aggregation diseases are still inadequate. The number of afflicted patients is increasing because of an aging population. One of the main hurdles for these diseases is the poor understanding of how protein aggregation processes are associated with toxicity. Compared with protein folding, protein misfolding is plastic and affords hard-to-target, conformational diverse, shape-shifting species. One of the key points in protein aggregation diseases is the multitude of structures, known as polymorphism, that appear because of the plasticity of misfolded proteins. Hence, we hypothesized that this is an important mechanism for disease phenotype and refer to these diseases in plural, e.g., Alzheimer’s diseases (Rasmussen et al., 2017; Liu et al., 2021).

### Protein Folding vs. Misfolding

**FIGURE 1:**
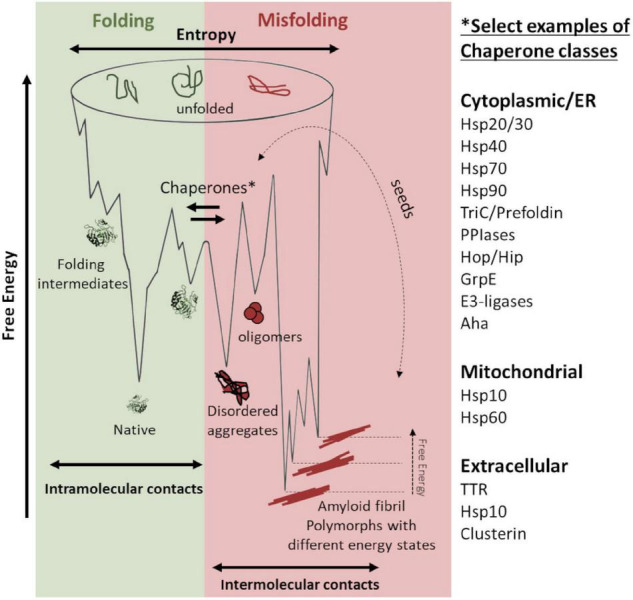
Schematic energy landscapes of protein folding (denoted in green) and misfolding and aggregation (denoted in red). Molecular chaperones keep proteins from misfolding at the intersection between the energy landscapes. There are hundreds of chaperones that facilitate protein folding and shift the landscape toward correct folding (denoted in green). Examples of chaperone classes are listed in the figure. Various amyloid fibril polymorphs have different stable structures, which are kinetically accessible and can propagate by seeded templating (denoted in dotted arrow). Seeding propagates misfolding in an antichaperone mechanism. This results in a tug-of-war between these two sides of the energy landscape: chaperones vs. antichaperones, which results in impaired proteostasis.

The protein folding process can be illustrated using an energy landscape, a depiction of the stability (free energy), entropy, and conformation (Figure 1, denoted in green). In this context, interactions are intramolecular, and under permissive conditions, the native state is thermodynamically favored (Anfinsen, 1973). For misfolding, aberrant intermolecular interactions stabilize a multitude of ordered conformational states, ultimately producing a “cloud” distribution of conformations referred to as amyloid fibril polymorphs (Figure 1, denoted in red). Interestingly, the polymorphs can replicate and hereby impose and template their structure by recruitment of new monomers separated by a kinetic barrier. In this study, seeds are catalyzing a nucleation-dependent polymerization reaction. These seeds act as antichaperones in driving propagated misfolding (Figure 1, red).

**FIGURE 2:**
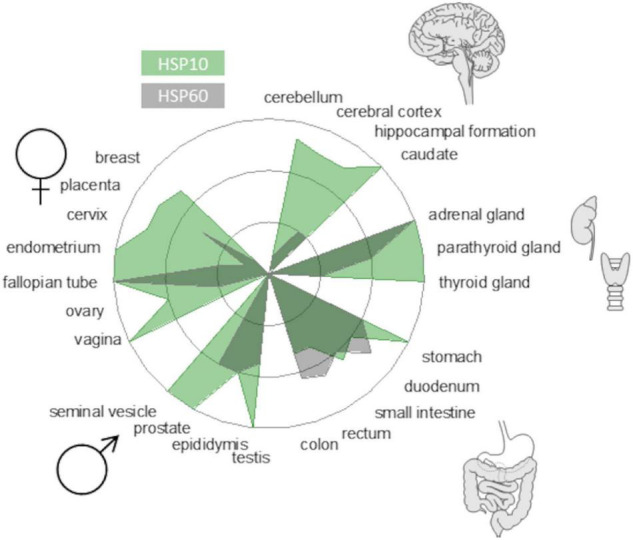
Protein abundance of HSP10 and HSP60 in different human tissues from the Human Protein Atlas (www.proteinatlas.org). The radar plot depicts levels from high to non-detectable. HSP10 is denoted in green and HSP60 is denoted in gray. Note that the abundance of HSP10 is very high in brain tissue implicating the importance of HSP10 for neurodegenerative diseases.

A prevailing hypothesis why protein aggregation diseases are associated with aging is that protein homeostasis (proteostasis) is less efficient in old age and becomes overwhelmed, resulting in a multitude of cellular problems. Consequently, understanding how the proteostasis balance is maintained or challenged can be an avenue for therapeutic interventions. In this study, we focused on molecular chaperone steering pathways (Figure 1, center) that govern productive protein folding, potentially modulate misfolding, and possibly mitigate neurotoxicity. There are several studies on chaperone influence on amyloid mitigation *in vitro* and in various model systems (Tittelmeier et al., 2020). Recently, these studies have also included human HSP60 (Marino et al., 2019; Vilasi et al., 2019) and the bacterial HSP60 GroEL, which have been shown to be very efficient in inhibiting amyloid fibril formation of Aβ *in vitro* (Fukui et al., 2016; Wälti et al., 2018). We have, for quite some time, been interested in the GroEL co-chaperone GroES. GroES has been shown to possess both holdase and unfoldase activity and also operate in the absence of GroEL during protein folding (Moparthi et al., 2014; Moparthi et al., 2016). In that context, HSP10, the human homolog of GroES, has some particularly interesting features. First, despite being an essential chaperone for mitochondrial proteostasis, HSP10 is also an extracellular protein. Many years ago, it was discovered that HSP10 is an early pregnancy factor expressed as a secreted protein within 24 h of gestation (Cavanagh and Morton, 1994). The biology behind this overexpression is still not fully understood but underscores that HSP10 can be abundant in circulation under stressed conditions. Second, data from the human protein atlas show that the HSP10 protein is vastly more abundant than HSP60 (Figure 2) despite sharing gene location, and HSP10 has its promoter in line with HSP60. This immense ubiquitous abundance, in particular in the brain (Figure 2), of HSP10 proposes more activities beyond the essential role as a co-chaperone for HSP60. Together, this suggests that HSP10 is an interesting but understudied chaperone, especially in the context of protein misfolding in the CNS. In this study, we investigated the effect of HSP10 on *in vitro* fibrillation kinetics of Aβ1-42, PrP, and fibril morphology.

## Materials and Methods

### HSP10 Purification

The purification protocol of HSP10 is based on the method described by Kamireddi et al. (1997). Plasmids containing synthetic genes for HSP10 homologs (ordered from Genscript and cloned at SciLifeLab) were transformed into *Escherichia coli* BL21 and grown on agar plates containing 50 μg/ml kanamycin. The agar plates were incubated overnight. The bacteria were cultured in 1 L LB containing 50 μg/ml kanamycin in 37°C until OD_600_ reached 0.4. The chaperones were induced with 0.5 mM isopropyl β-D-1-thiogalactopyranoside (IPTG), and the cultures were incubated at 16°C for 16 h. The cultures were harvested by centrifugation at 4,000 *g* for 30 min. The bacteria were resuspended in 30 mM imidazole buffer pH 8. Lysis of bacteria was carried out with sonication with the settings (30 s on, 30 s off with an amplitude of 30% for a total on time of 2 min). The cell debris was pelleted by centrifugation at 4,000 *g* for 30 min. The protein lysate was heated to 80°C for 20 min and then centrifuged at 15,000 *g* for 10 min. The supernatant was extracted and centrifuged for 20 min at 12,000 *g*. The supernatant was placed on an equilibrated immobilized metal affinity chromatography (IMAC) column. Furthermore, 30 mM imidazole buffer of pH 8 was added in the washing step, and the chaperones were eluted in 300 mM imidazole of pH 7. The chaperones were dialyzed in 1 L phosphate-buffered saline (PBS) buffer containing 10 mM EDTA pH 7.4 for 1 h; the chaperones were then placed on dialysis in PBS pH 7.4 containing 0.02% NaN_3_ overnight. Concentration was determined with a spectrophotometer using the formula (Abs[280] − Abs[300])/ε to minimize background contribution. The extinction coefficient used was 2,980 M^–1^cm^–1^ for GroES, 5,960 M^–1^cm^–1^ for HuHSP10 and 1,490 M^–1^cm^–1^ for DrHSP10. All constructs have an extra tyrosine placed in the TEV protease site (Supplementary Figure 2). The concentrations were also verified with the Bradford assay, with bovine serum albumin (BSA) as a standard. Purity and identity were assessed with Coomassie-stained SDS-PAGE gels and with matrix-assisted laser desorption/ionization-time of flight (MALDI-TOF) (Supplementary Figure 1).

### MALDI-TOF

HSP10 homologs were dialyzed in dH_2_O. The chaperones were mixed with sinapinic acid and loaded on a MALDI-TOF sample target plate. Transthyretin (TTR) monomers, dimers, trimers, and tetramers were used to calibrate the MALDI-TOF (monomeric TTR m/z = 13,892.60). Spectra were obtained using an UltrafleXtreme MALDI system (Bruker Daltronics).

### BiP Purification

The purification protocol for BiP was based on the method described by Sörgjerd et al. (2006) using the same plasmid. The plasmid containing the gene for BiP was transferred into *E.coli* BL21 cells and cultured on agar plates containing 100 μg/ml ampicillin overnight, at 37°C. Bacteria were cultured in 1 L LB containing 100 μg/ml ampicillin at 37°C until OD_600_ reached 0.6. BiP protein production was induced with 1 mM IPTG, and the culture was incubated at 37°C for 4 h. The bacteria were harvested with centrifugation at 4,000 *g* for 30 min. The pellet was resuspended in dH_2_O and stored at −20°C. The cells were resuspended, and the buffer was replaced with 30 mM imidazole buffer, and protease inhibitors were added. Cells were lysed with a cell disruptor with a pressure 25 kpa, and the lysate was centrifugated at 16,000 *g* for 30 min. The supernatant was placed on the equilibrated IMAC column. Furthermore, 30 mM imidazole buffer of pH 8 was added as a washing step, and the chaperone was eluted in 300 mM imidazole of pH 7. The protein was dialyzed first in 1 L PBS with 10 mM EDTA for 1 h and then in 1 L PBS overnight. 10 mM ATP was then added to the dialyzed protein to promote the release of potential bound bacterial peptides. The solution was placed in a cold room on low shaking for 1 h. BiP was then placed on an IMAC again, eluted using 300 mM imidazole, dialyzed in 1 L PBS of pH 7.4 with 10 mM EDTA for 1 h, and then in 1 L PBS pH 7.4 containing 0.02% NaN_3_ overnight. Concentration was determined with a spectrophotometer using the formula (Abs[280] − Abs[300])/ε to minimize background contribution. The extinction coefficient used for BiP was 27,850 M^–1^cm^–1^. Purity and identity were assessed with Coomassie-stained SDS-PAGE gels.

### Aβ1–42 Purification

Aβ1–42 was either recombinantly produced following the protocol from Sandberg and Nyström (2018) or purchased from rPeptide. Aβ1–42 plasmid (AβM1–42) was transformed into *E. coli* plysS and cultured on agar plates containing 50 μg/ml ampicillin and 25 μg/ml chloramphenicol overnight. Aβ1–42 as cultured in 37°C in 2 L LB cultures and with 50 μg/ml ampicillin and 25 μg/ml chloramphenicol until OD600 reached 0.6. The cultures were then induced with IPTG to a final concentration of 0.5 mM. The cultures were incubated at 37°C for 4 h and then harvested by centrifugation 4,000 *g* for 30 min. The pellets were then resuspended in mqH_2_O and stored at −20°C overnight. The pellet was reharvested in by centrifugation at 16,000 *g* for 10 min, and mqH_2_O was replaced with 10 mM Tris and 1 mM EDTA pH 8 (buffer A). The pellet was resuspended and sonicated with the settings 30 s on, 30 s off with an amplitude of 30% for a total time of 2 min. The sonicated cells were then centrifuged at 18,000 *g* for 10 min. The supernatant was extracted, and the pellet was resuspended in buffer A. This procedure was repeated three times. After the final centrifugation, the pellet was resuspended in 8 M urea, 10 mM Tris 1 mM EDTA pH 8, and sonicated with the settings 30 s on, 30 s off with an amplitude of 30% for a total time of 2 min. The mix was then centrifuged with the settings 18,000 *g* 10 min and 4°C, and the supernatant was collected. The urea lysate was added to diethylaminoethyl (DEAE) beads and incubated for 20 min. The solution was filtered through the beads. Furthermore, 40 ml wash buffer (10 mM Tris, 1 mM EDTA, 25 mM NaCl pH 8) was added and filtered through the beads. A total of 40 ml low salt buffer (10 mM Tris, 1 mM EDTA, 150 mM NaCl pH 8) was added and filtered through the beads; 40 ml high salt buffer (10 mM Tris, 1 mM EDTA, 500 mM NaCl pH 8) was added and filtered through the beads. The low-salt and high-salt fractions were placed on dialysis in 8 L of 2 mM NaOH for 1 h. The dialysis buffer was then exchanged to 8 L of 2 mM NaOH, and dialysis was continued overnight. The low-salt and high-salt fractions were concentrated in centriprep tubes with a cutoff at 3,000 Da, and the concentrated protein was lyophilized.

### Transthyretin Purification

Transthyretin WT was purified as previously described (Groenning et al., 2011), and the concentration was determined by using, ε = 77,600 M^–1^cm^–1^, at 280 nm, and 55 kDa molecular mass for native tetrameric TTR.

### HuPrP90-231 Purification

HuPrP was purified as previously described (Sandberg and Nyström, 2018). In short, protein was expressed overnight in *E. coli* BL21/DE3 cells. Cells were resuspended in buffer containing 6 M GuHCl with reducing agent glutathione and lysed by sonication. Cleared cell lysate was applied to NiNTA agarose beeds and protein was stepwise refolded and disulfide bond reformed before elution with 500 mM imidazole. Subsequent size exclusion chromatography on a Superdex 75 column (GE-Healthcare) enabled collection of natively folded monomeric HuPrP in 50 mM Na_2_HPO_4_ (pH 7.4), 100 mM NaCl, 50 mM KCl. Protein concentration was determined using ε = 27,515 M^–1^cm^–1^, at 280 nm, and 18 kDa molecular mass.

### Fibrillation of Aβ1–42 With Chaperones Present

Regardless of the source, the lyophilized Aβ1–42 was resuspended in 6 M GuHCl and centrifuged for 1 h at 18,000 *g*. The protein was purified on a Superdex 75 10/300 column (GE-Healthcare) to isolate fresh monomer prior to fibrillation and eluted in PBS pH 7.4 containing 0.02% NaN_3_. Concentration was determined using the spectrophotometer using the equation (Abs[280 nm] – Abs[300 nm])/1,490 M^–1^cm^–1^. The fibrillation of Aβ1–42 was conducted in quiescent conditions in either non-treated 96-well half-area plates (Corning 3880) or non-binding 96-well half-area plates (Corning 3881) at 37°C. Furthermore, 10 μM thioflavin T (ThT) was present during fibrillation, and the fluorescence was measured with the excitation of 440 nm and emission of 480 nm. Depending on the experiment, the kinetics was either measured in a Tecan infinite M1000 Pro or a BMG Clariostar fluorescence plate reader. Kinetic data were fitted to sigmoidal function as described before (Gade Malmos et al., 2017); the half-times of fibrillation were obtained for each fitted trace when the normalized ThT fluorescence intensity reached 0.5.

### Amylofit

The modeling of Aβ1–42 aggregation kinetics was conducted with the program Amylofit, developed by Meisl et al. (2016). Aggregation kinetics of Aβ1–42 and chaperones conducted in non-binding half area 96-well plates (Corning 3881) were used for the Amylofit analysis. A secondary nucleation-dominated and unseeded model was used to fit the ThT traces since this is the described model for Aβ1–42 aggregation under quiescent conditions (White et al., 2013). The joint k_*n*_k_+_ and k_2_k_+_ constants were separately tested as free fitting parameters. During the k_*n*_k_+_ fit, k_2_k_+_ was globally fitted to all ThT traces, and during the k_2_k_+_ fit, k_*n*_k_+_ was globally fitted to all ThT traces.

### Fibrillation of PrP With Chaperones Present

Fibrillation of PrP was performed as described previously (Sandberg and Nyström, 2018). Six replicates of each experimental condition were prepared to compensate for the stochastic kinetics displayed by HuPrP when fibrillated under native conditions. The concentration of HuPrP and chaperone was 5 and 2.5 or 0.25 μM, respectively. Fibrillation was performed in a sealed 96-well plate (Corning 3880) at 37°C and high-intensity linear shaking in a Tecan Saphire 2 plate reader. Kinetics was monitored by fluorescence intensity at 480 nm using a final concentration of 2 μM ThT. Kinetic data were fitted to sigmoidal function as described before (Gade Malmos et al., 2017), and the half-times of fibrillation were obtained for each fitted trace when the normalized ThT fluorescence intensity reached 0.5.

### Transmission Electron Microscopy

5 μl fibrillated Aβ1–42 was placed on a TEM grid (Carbon-B, Ted Pella Inc.) and incubated for 1 min. The sample was removed by filter paper blotting, and the grid was washed with 5 μl dH_2_O. Furthermore, 5 μl of 2% uranyl acetate in water was added as negative stain and incubated for 30 s and then dried. Micrographs were obtained using a JEOL 1,230 transmission electron microscope equipped with a CCD camera.

### Hepta-Formyl Thiophene Acetic Acid and Quadro-Formyl Thiophene Acetic Acid Staining

5 μl of Aβ1–42 fibrils were mixed with qFTAA and hFTAA to a final concentration of 1,000 nM qFTAA and 500 nM hFTAA. The fibrils were incubated overnight. Additionally, 5 μl stained fibrils were placed on a microscope objective slide and were dried at room temperature, mounted with Dako fluorescence mounting medium. Hyperspectral images were obtained using a Leica DM6000B epifluorescence microscope equipped with long band-pass filters and a SpectraView system (Applied Spectral Imaging). A SpectraView 4.0 and a Spectra-Cube (inferometric optical head SD 300) module with a cooled CCD camera, and a D436/10x; E475LPv2; 455DCLP emission bandpass filter set were used. The data were processed with the SpectraView 3.0 EXPO software. Emission spectra were collected in an interval of 460–750 nm with the highest spectral resolution settings (“gas-line settings”). Images of fibrils were obtained using the 40× objective.

### HSP10 Chaperone Binding to Immobilized Fibrils

Aβ1–42 fibrils were diluted in 15 mM Na_2_CO_3_, 35 mM NaHCO_3_ buffer (pH 9.6) and horn sonicated for 6 min, 1 min on 1 min off with an amplitude of 30%. A high binding plate (Corning 9018) was coated with 50 μl of 2.5 μg/ml Aβ1–42. The plate was shake-incubated overnight at 8°C. Washing was conducted with PBS pH 7.4 with 0.05% Tween. The plate was blocked with PBS 7.4 containing 2% BSA and shake-incubated for 1 h at room temperature. Chaperones were diluted in 2% BSA to the appropriate concentration and loaded into the wells. The plate was then shake-incubated at room temperature for 2 h. Polyclonal rabbit anti 6× HIS (Ab1187) was added to the wells, and the plate was shake-incubated at room temperature for 1 h. 3,3′,5,5′-Tetramethylbenzidine substrate was added to the well, and the reaction was terminated after 30 min using 0.18 M H_2_SO_4_. The absorbance at 450 nm was measured in a Tecan infinite M1000 Pro plate reader. Standard curves of pure chaperones directly deposited in a high binding plate (Corning 9018) (concentration of 0–100 nM) were used for reference to estimate the amount of HSP10 bound to fibrils. The data were fitted to a dose–response function in GraphPadPrism v9.1, and the final Abs signal of each fibril binding curve was compared with the fit of the standard curve.

## Results

We selected HSP10s from human, *E. coli* (GroES), and *Drosophila melanogaster*. Human HSP10 (HuHSP10) was selected as a homologous system for human amyloid disease, GroES as a well-studied reference chaperone, and *Drosophila* HSP10 (DrHSP10) due to our experience with transgenic models for human amyloid diseases in the fly suffering from neurodegeneration (Berg et al., 2009; Berg et al., 2010; Jonson et al., 2015; Jonson et al., 2018; Jonson et al., 2019; Sandberg et al., 2020). Our main system for fibril formation was Aβ1–42 for its association with Alzheimer’s disease. Aβ1–42 also represents a canonical protein for fibril formation kinetics and fibril polymorphism (Fändrich et al., 2018). We also included TTR, and the HSP70 chaperone BiP as reference chaperones previously shown to have amyloid inhibiting effects on several proteins, including Aβ. Primarily, suppression of Aβ1–40 fibrillation rates has been reported for TTR (Li et al., 2013; Nilsson et al., 2018; Ghadami et al., 2020).

### Aβ1–42 Fibrillation Kinetics

**FIGURE 3:**
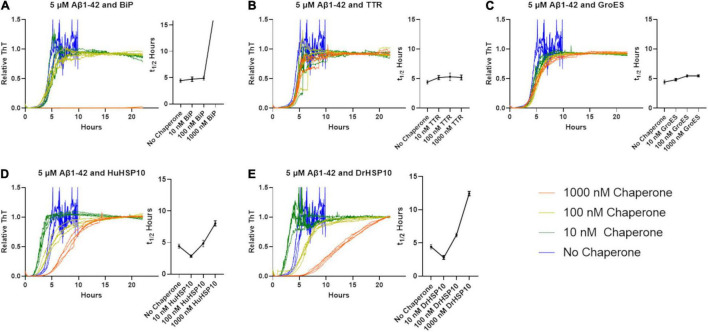
Normalized Thioflavin T (ThT) traces of Aβ1–42 fibrillation (5 μM) when chaperones are present at various concentrations. Traces are colored according to the legend in the figure. Corresponding halftimes of fibril conversion, t_1/2_ (hours), for each concentration are shown to the right in each panel. **(A)** Aβ1–42 and BiP, **(B)** Aβ1–42 and TTR, **(C)** Aβ1–42 and GroES, **(D)** Aβ1–42 and HuHSP10, and **(E)** Aβ1–42 and DrHSP10. The assay was conducted in quiescent conditions at 37°C in non-treated 96-well plates.

The effect of HSP10s on fibrillation rates was investigated with recombinant Aβ1–42. Fibrillation of Aβ1–42 in the presence of HSP10 homologs for *E. coli* (GroES), *Drosophila* HSP10 (DrHSP10), and human HSP10 (HuHSP10) was conducted with a constant concentration of Aβ1–42 and varying substoichiometric concentrations of chaperones. As references under identical conditions, we included the known fibrillation chaperones human TTR (55 kDa) and the ER resident HSP70, Grp78/BiP (70 kDa). Fibrillation kinetics were monitored with the amyloid fluorescent probe ThT (Figures 3A–E). The lag-time for fibril formation of Aβ1–42 is approximately 4 h under these conditions (5 μM, PBS buffer, pH 7.4, non-treated 100 μl 96-well plates). GroES and TTR did not substantially influence the Aβ1–42 fibrillation kinetic profiles in our experimental setup (Figures 3B,C). A very minor increase in half time of conversion (t_1/2_) was observed as a correlation with increased chaperone concentration (Figures 3B,C). Tetrameric-folded TTR WT being inefficient in inhibiting fibrillation of Aβ1–42 is consistent with previous reports (Cao et al., 2020). The presence of HuHSP10, DrHSP10, and BiP markedly delayed fibrillation for Aβ1–42 at the highest concentration of chaperones (Figures 3A,D,E). The suppression of fibrillation was, however, different for the chaperones investigated. HuHSP10 and DrHSP10 delay the aggregation when present in a ratio of 0.2 (1 μM) and 0.02 (100 nM) (Figures 3D,E), while BiP showed complete inhibition at a ratio of 0.2 (1 μM) over 24 h, but no observable effect when the concentration of the chaperone was lower (Figure 3A). BiP has been proposed to bind as a holdase for monomeric misfolded proteins and work as a molecular shepherd forming complexes with oligomers of misfolded proteins allowing it to mitigate fibrillation at substoichiometric concentrations (Sörgjerd et al., 2006) as herein by efficient suppression at 0.2 BiP per Aβ1–42 molecule. Interestingly, the effect of HuHSP10 and DrHSP10 was concentration dependent in a different manner than BiP. The suppression effect in terms of prolonged t_1/2_ was more pronounced for DrHSP10 compared with HuHSP10 (cf. Figures 3D,E). Paradoxically, fibrillation of Aβ1–42 in the presence of very low concentrations (10 nM) of DrHSP10 and HuHSP10 showed a decreased lag-time, leading to faster onset of fibrillation. Note the V-shaped t_1/2_ plots as a function of chaperone concentration (Figures 3D,E).

### Aβ1–42 Fibril Morphology

**FIGURE 4:**
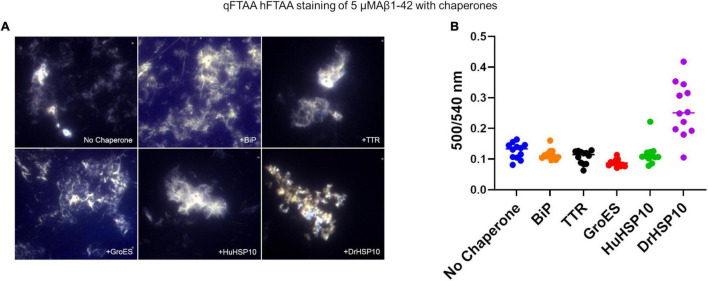
Amyloid staining with luminescent conjugated oligothiophenes (LCOs) of Aβ1–42 fibrils formed in the absence or presence of different chaperones. **(A)** Representative hyperspectral micrographs of fibrils stained with the LCOs hepta-formyl thiophene acetic acid (hFTAA) and quadro-formyl thiophene acetic acid (qFTAA). **(B)** Plot of intense regions of interest (ROIs) of qFTAA:hFTAA emission intensity ratio (500/540 nm) from the hyperspectral micrographs. Complete set of micrographs in Supplementary Figure 3. Concentrations during fibrillation: Aβ1–42, 5 μM, chaperones 1 μM.

To make a comparative conformational analysis of Aβ1–42 fibrils formed in the absence and presence of chaperones, we performed hyperspectral fluorescence microscopy analysis of fibrils incubated without ThT for 48 h and thereafter co-stained with the conformation sensitive luminescent conjugated oligothiophenes (LCOs) qFTAA and hFTAA (Nyström et al., 2013; Rasmussen et al., 2017; Liu et al., 2021). Aβ1–42 fibrils formed *in vitro* are usually dominated by hFTAA fluorescence as is displayed by low ratio values in the ratio plot of qFTAA/hFTAA fluorescence (Figure 4B). Even though BiP efficiently inhibited fibril formation kinetics, fibrils were visible in the microscope likely due to the long incubation time used in this study (48 h). The fibrils formed in the presence of BiP showed the same LCO signal as unchaperoned fibrils, the same was observed for TTR (Figure 4B). Neither BiP nor TTR chaperone changed the microscopic appearance of the fibrils (Figure 4A). Similarly, GroES did not change overall qFTAA and hFTAA spectra nor the microscopic fibril morphology (Figures 4A,B). For HuHSP10, a non-significant increased qFTAA contribution was observed (Figure 4B). Notably, the qFTAA contribution was significantly increased for fibrils formed in the presence of DrHSP10 (Figure 4B). Aβ1–42 fibrils formed in the presence of DrHSP10 showed a different microscopic appearance with prominent granular aggregate clusters rather than extended fibrils (Figure 4A and Supplementary Figure 3).

**FIGURE 5:**
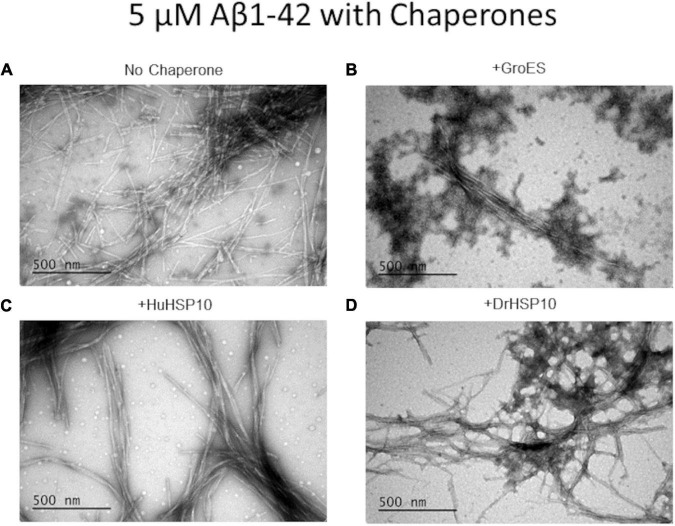
Ultrastructure of Aβ1–42 fibrils formed in the absence or presence of different HSP10 chaperones, imaged by transmission electron microscopy (TEM). **(A)** Aβ1–42 no chaperone, **(B)** Aβ1–42 + GroES, **(C)** Aβ1–42 + HuHSP10, and **(D)** Aβ1–42 + DrHSP10. Concentrations during fibrillation: Aβ1–42, 5 μM, chaperones 2.5 μM. Presence of HSP10 appeared to promote multifilament bundled fibrils.

The Aβ1–42 fibril morphology of fibrils formed in the presence of HSP10s was investigated with TEM. Aβ1–42 without chaperone yielded variable-sized mixture of long and short fibrils (Figure 5A), while the presence of HuHSP10 gave rise to thicker, elongated, and bundled fibrils (Figure 5C). The presence of both GroES and DrHSP10 during Aβ1–42 fibrillation promoted co-localized extended bundled fibrils with amorphous aggregates (Figures 5B,D). However, Aβ1–42 fibrils formed in the presence of GroES showed amorphous aggregates around the fibrils, while DroHSP10 promoted amorphous aggregation at the end of the fibrils (Figures 5B,D). Overall, the TEM data were suggestive of HSP10 influence of Aβ1–42 fibril polymorphism.

### Structural Basis for HSP10 Fibril Binding and Modulation of Fibril Formation

**FIGURE 6:**
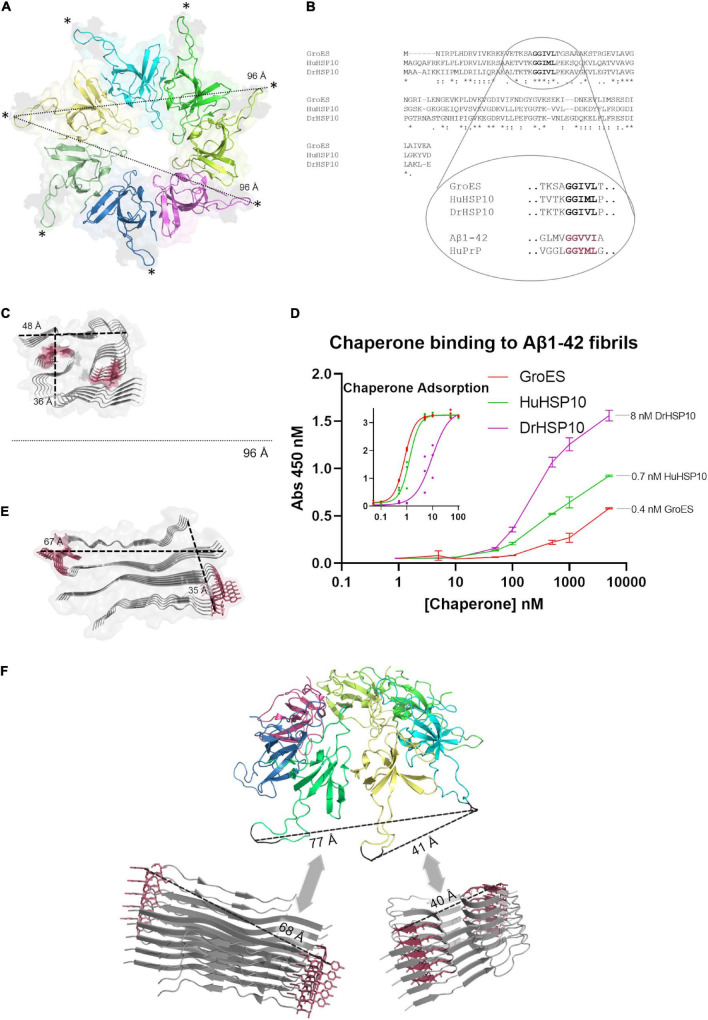
Structural comparisons of HSP10s and amyloid filaments. **(A)** Ribbon diagram of the cryo-EM structure of HuHSP10 bound to human HSP60 (PDB code: 6MRC) (Gomez-Llorente et al., 2020). Each subunit in the heptamer is depicted in different colors. The maximum cross-distance between the mobile loops (highlighted with *) when structured and bound to HSP60 is 96 Å. **(B)** Multiple sequence alignment of GroES, HuHSP10, and DrHSP10 highlighting the conserved mobile loop sequence 29–33 (human numbering) GGIM(V)L. The mobile loop sequence is similar to amyloidogenic segments of Aβ (GGVVI 37–41) and PrP (GGYML 126–130) highlighted in red. **(C)** Structure of a filament type I polymorph from human brain Aβ1–42 (PDB code: 7Q4B) (Yang et al., 2022) drawn to scale with **(A)** and the core segments 37–41 shown in red. **(D)** Relative HSP10 binding affinity and saturation to sonicated immobilized Aβ1–42 fibrils. Error bars represent ± SD of triplicates. Inset [same x- and y-axes as in panel **(D)**] shows a standard curve of HSP10 (0–100 nM) to estimate the concentration of HSP10 bound at the highest concentration of applied chaperone in the main figure **(D)**. **(E)** Structure of an amyloid filament of PrP106–145 (PDB code: 6UUR) (Glynn et al., 2020). The amyloidogenic segment 126–130 is highlighted in red. Drawn at scale with **(A,C)**. **(F)** Speculative structural illustration of how a heptameric molecule of HSP10 could recognize fibril ends of a Aβ1–42 (bottom right) and PrP106–145 (bottom left) by multiple binding of mobile loops fitting within variable distances depending on fibril structure. All protein structure images were made with PyMOL.

We asked the question how HSP10s recognize amyloid fibrils? The structure of HSP10 comprises a homo-heptamer forming a dome with seven tangling arms composed of mobile loops (Figure 6A). The mobile loop edges are recognition motifs for binding to a hydrophobic patch within each subunit at the top of the apical domains of the HSP60 heptamer fitting like a cogwheel (Gomez-Llorente et al., 2020). The primary sequence of the evolutionary distant HuHSP10, DrHSP10, and GroES is rather variable, but the edge of the mobile loop is the most well-preserved segment within the protein sequence (Figure 6B). This segment GGIML/GGIVL is similar to a C-terminal sequence of Aβ1–42, 37–41 GGVVI. It is well established that fibril formation is promoted by sequence similarity. Multiple recognition elements within the heptameric HSP10 structures could provide high avidity toward a fibril end, rather than to the fibril surface. Amino acids 37–41 in the Aβ1–42 sequence is involved in filament core packing in all Aβ1–42 fibril filament polymorphs structurally determined at high resolution (Figure 6C) (Fändrich et al., 2018; Yang et al., 2022). The maximum distance between the mobile loops in the HSP10 heptamer (bound to HSP60) is 96 Å, which is clearly wide enough to embrace over a cross-section (fibril end) (Figure 6C). A bundle of multiple filaments of Aβ1–42 would also fit this maximal distance. We performed a binding assay on immobilized sonicated Aβ1–42 fibrils and note a fair affinity (K_*d*_ ∼ 500–1,000 nM) and variable apparent binding saturation values at the highest assayed HSP10 concentration (5,000 nM) (Figure 6D). It is clear from the graph that the binding affinity and saturation (B-value) on immobilized sonicated Aβ1–42 fibrils was in the following order DrHSP10 > HuHSP10 > GroES (Figure 6D). Direct deposition of the respective HSP10s at different concentrations (0–100 nM concentration directly in the binding plate) allowed us to estimate the number of chaperones per Aβ1–42 monomer bound to the fibrils (Figure 6D and inset). From this comparison, one DrHSP10 molecule bound per 70 Aβ1–42 monomers, one HuHSP10 per 800 monomers, and one GroES per 1,400 monomers. Such low B-values support the hypothesis that the HSP10 chaperones bind to fibril ends, which would correlate well with our structural interpretation. Notably, in a previous report on the monoclonal antibody gantenerumab, it was estimated that one antibody molecule bound per 44 Aβ1–42 monomers in the fibril, hence proposed by Linse and co-workers to target fibril ends, consistent with the observed kinetic profile of this antibody in inhibiting fibril growth (Linse et al., 2020).

### Fibrillation Acceleration

**FIGURE 7:**
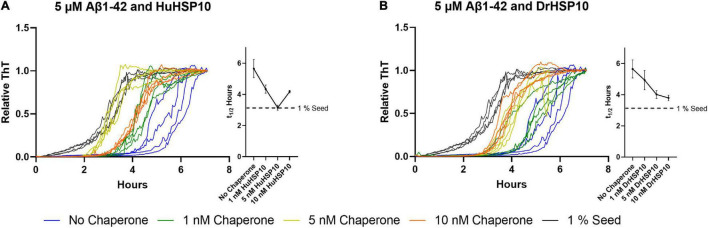
Normalized ThT traces of Aβ1–42 fibrillation (5 μM) when chaperones are present at low concentrations as well as Aβ1–42 fibrillated in the presence of 1% preformed fibril seeds. Traces are colored according to the legend in the figure. Corresponding halftimes of fibril conversion, t_1/2_ (hours), for each concentration are shown to the right in each panel. **(A)** Aβ1–42 and HuHSP10 and **(B)** Aβ1–42 and DrHSP10. In this concentration interval, both HuHSP10 and DrHSP10 accelerate the fibrillation of Aβ1–42 compared with the spontaneous fibrillation of Aβ1–42 alone. The assay was conducted in quiescent conditions at 37°C in non-treated 96-well plates.

Interestingly, fibril formation appeared to be accelerated at very low HSP10 concentrations (10 nM for DrHSP10 and 10 and 100 nM for HuHSP10) (Figures 3D,E). We followed up this observation with additional experiments of low concentrations of HuHSP10 and DrHsp10 (0–10 nM) in comparison with the acceleration effect induced by 1% preformed Aβ1–42 fibril seeds. These experiments showed that the acceleration effect was obvious already at the interval 1–5 nM of HSP10 (Figures 7A,B). While the acceleration effect on shortening the lag-phase was not as efficient at 1% preformed fibril seeds (corresponding to 50 nM Aβ1–42 on a monomer basis), the effect was noticeable and most efficient for HuHSP10 peaking at 5 nM providing a t_1/2_ of fibrillation at the same level as 1% fibril seeds (cf. Figures 7A,B). Notably, the same acceleration effect was previously observed for human HSP60 at similar low stoichiometries of chaperone to Aβ1–42 but not by GroEL (Vilasi et al., 2019). The acceleration of fibrillation at low concentration of HSP10 could be explained by HSP10 grabbing and stabilizing a fibril nucleus initiating fibrillation (Figure 6F). This acceleration effect was not observed for antibodies believed to bind to fibril ends (Linse et al., 2020) suggesting that HSP10s by virtue of their multiple binding sites organized in a symmetric heptamer are responsible for this effect. This hypothesis is consistent with the extensive length of fibrils and the bundling of fibrils observed by TEM for all three HSP10 chaperones, but in particular, by HuHSP10 (Figure 5C). A C-terminal segment involving AβV40 and AβI41 was shown to be involved in binding of Aβ1–42 to GroEL (Wälti et al., 2018) as expected from the corresponding sequence in the mobile loop edge of GroES. While the acceleration effect of GroES was not observed, as was evident for DrHSP10 and HuHSP10s, the binding efficacy toward Aβ1–42 fibril ends was also lower for GroES (Figure 6D). Hence, besides the mobile loop sequence, the size and dynamic properties of the HSP10 heptamer appear to play a role in modulating Aβ1–42 fibrillation.

### Amylofit Analysis

**FIGURE 8:**
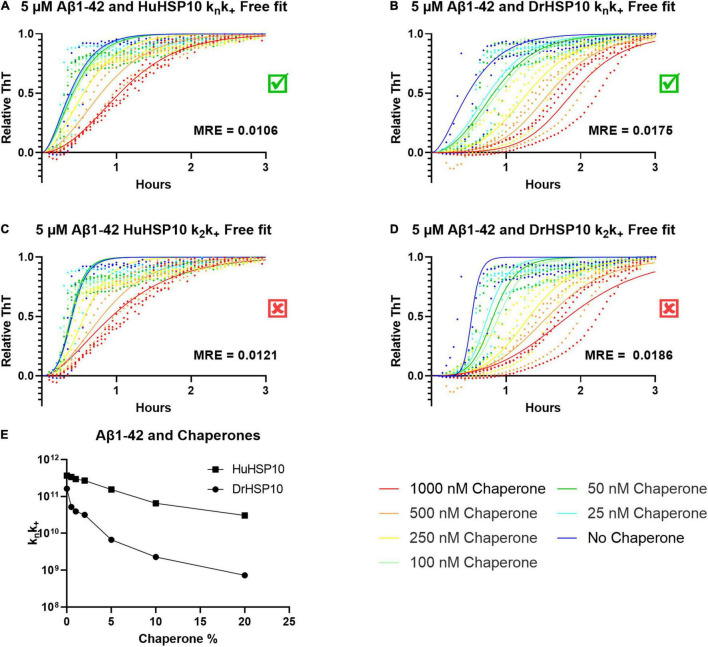
ThT kinetics for 5 μM Aβ1–42 with the chaperones HuHSP10 and DrHSP10 present at various concentrations with model curves fitted using Amylofit. **(A,C)** Fitted data for HuHSP10, **(B,D)** fitted data for DrHSP10. Both chaperones fit best to free fitting parameters k_*n*_k_+_, as shown by mean squared residual error (MRE) of the global fits, i.e., chaperones were mostly affecting primary nucleation and elongation and worse fits for secondary nucleation (k_2_) and elongation. **(E)** k_*n*_k_+_, as a function of substoichiometric chaperone concentration, to compare HuHSP10 and DrHSP10, respectively. DrHSP10 was more efficient than HuHSP10 in suppression of fibrillation of Aβ1–42. Each dot represents an average of four replicates; the assay was repeated three times in a 96 well non-binding plate.

Does fibril binding of HSP10 correlate with the kinetics? From the kinetics of Aβ1–42 fibrillation, we observed that at the higher concentrations of DrHSP10 and HuHSP10 chaperones, the lag time is prolonged, and the growth phase was slowed down (Figures 3D,E). Further experiments were performed under conditions allowing the Amylofit analysis. The Amylofit analysis allows us to model the data for an approximation of which kinetic steps were influenced by HuHSP10 and DrHSP10. To accommodate the assay for this analysis, we switched to non-binding plates where the lag phase of unchaperoned Aβ1–42 fibrillation is 10 min (Figure 8). To identify how the chaperones modulate the aggregation of Aβ1–42, the kinetic models were fitted to the normalized raw data. The aggregation of Aβ1–42 in the absence of chaperones was assumed to be dominated by secondary nucleation (White et al., 2013). Fitting was conducted with the free parameter either being the k_*n*_k_+_ or k_2_k_+_, applying models with free fitting of primary or secondary nucleation and elongation constants.

The best overall fit was observed for fitting of k_*n*_k_+_ (Figures 8A,B). Hence, the data suggest that the effect at elevated concentrations of DrHSP10 and HuHSP10 chaperones mainly reduced primary nucleation and inhibited elongation in a concentration-dependent manner (Figures 8A,B,E). Notably, DrHSP10 had a more pronounced inhibitory effect on Aβ1–42 primary nucleation and elongation than HuHSP10 (Figure 8E).

### PrP Fibrillation Kinetics

**FIGURE 9:**
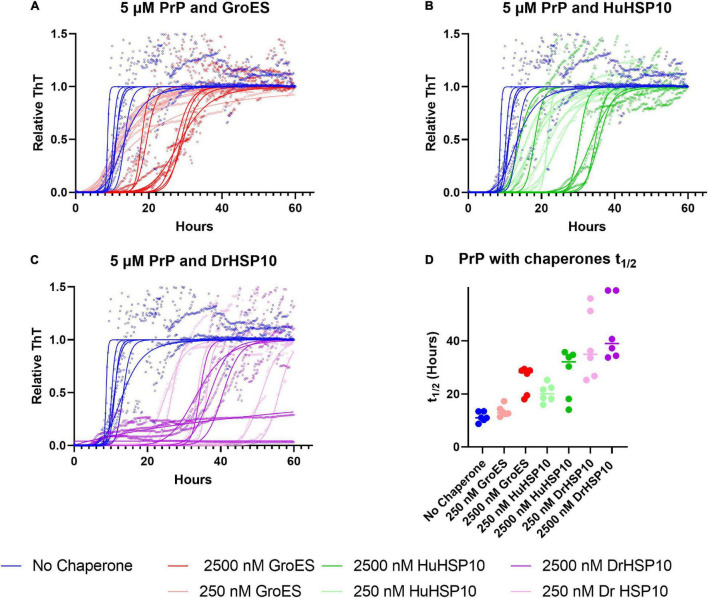
PrP fibrillated in the absence (denoted in blue) and presence of HSP10 chaperones. **(A)** GroES (denoted in red) and PrP, **(B)** HuHSP10 (denoted in green) and PrP, and **(C)** DrHSP10 (denoted in magenta) and PrP. The assay was conducted with shaking at 37°C in a non-treated 96-well plate. Concentrations during fibrillation: HuPrP90–231: 5 μM, chaperones: 2.5 or 0.25 μM. **(D)** Half-time of conversion (t_1/2_ in hours) in the absence and presence of the different chaperones. The plot illustrates concentration dependence and different activities in mitigating PrP fibrillation, showing the following order of effectiveness: DrHSP10 > HuHSP10 > GroES.

We next assayed fibrillation of HuPrP90–231 to interrogate the HSP10 chaperone activity on another amyloidogenic system associated with ND. HuPrP90–231 can be readily fibrillated under close to physiological conditions (5 μM PrP, neutral pH, and 37°C) albeit at intense shaking (Nyström et al., 2012; Rodrigo et al., 2016; Sandberg and Nyström, 2018). Under these conditions, HuPrP90-231 fibrillates with a half-time of approximately 10 h (Figures 9A–D). When HSP10 chaperones (Figures 9A–C) were present (2.5 or 0.25 μM), a delay of PrP fibrillation was observed. At the high concentration of chaperone, GroES, HuHSP10, and DrHSP10 all suppressed the aggregation of PrP by prolonging the lag phase and increased the growth phase substantially increasing the half-time of conversion to >30 h (Figure 9D). Notably, PrP contains an amyloidogenic sequence with high homology to the edge of the mobile loops of HSP10s (Figure 6B). HuPrP90–231 contains the sequence 126–130 GGYML. A recently described HuPrP fragment fibril structure polymorph shows that the 126–130 segment is embedded in the fibril structure forming an integral part of the in-register parallel β-arches comprising the fibril core of the amyloid filament (Figure 6E) (Glynn et al., 2020). We, hence, speculated that the chaperoning effect on PrP is also mediated by fibril end binding, and as in the case with Aβ1–42, the HSP10 seven mobile loops (96 Å apart) readily fit across filaments of PrP fibril ends (Figure 6E). Interestingly, also for PrP, DrHSP10 was most efficient in suppressing fibril formation (Figure 9D). These data together with the results from Aβ1–42 support the notion that HSP10 fibril chaperoning can be a general function, and that the mobile loops are the active sites for HSP10 fibril chaperoning. We also noticed that while the effect on t_1/2_ is negligible for GroES at low concentrations, it is obvious looking at the kinetic traces that it showed a shortened lag time while a less steep growth phase (Figure 9A), which together does not alter the t_1/2_ (Figure 9D). This was not observed for DrHSP10 and HuHSP10. Hence, it appears that the acceleration effect at low chaperone to substrate ratios of fibrillation is chaperone and substrate dependent because for PrP the acceleration effect was limited to GroES, which was instead limited to DrHSP10 and HuHSP10 for Aβ1–42.

## Discussion

**FIGURE 10:**
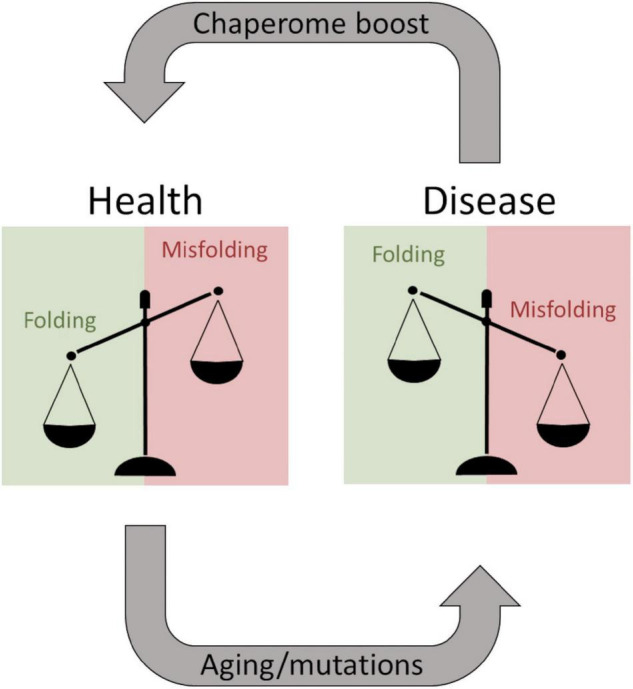
The balancing act of proteostasis. As long as chaperones efficiently keep proteins from misfolding a patient remains healthy. Aging and predisposing mutations facilitate protein misfolding shifting the balance and overwhelming the chaperome, resulting in degenerative diseases. These systems are potentially druggable to boost the chaperome to exacerbate the healthy situation during aging, and theoretically, this can be a therapeutic strategy even after disease onset.

Neurodegenerative diseases (NDs) represent a significant cause of death in the world, and there are no cures available for NDs. Accumulation of aggregated misfolded proteins is a common hallmark of NDs suggesting impaired proteostasis leading to cellular damage and collapse. Especially, the aging brain is subjected to impaired proteostasis exacerbating the risk for Alzheimer’s disease. Aβ aggregates are associated with Alzheimer’s disease and are the main target for disease-modifying therapies such as the recently approved Aducanumab (Sevigny et al., 2016). The development of monoclonal antibodies successfully targeting Aβ aggregates has also stimulated research into other aggregation modulating pharmaceuticals. Chaperone therapy is being considered as an avenue to fill this gap and to rebalance brain proteostasis during Aβ aggregation (Figure 10). The Bri2 BRICHOS domain is one of the most well-studied examples (Cohen et al., 2015; Buxbaum and Johansson, 2017), but aggregation of Aβ can be modulated by several different chaperone proteins.

HSP10 is a well-known co-chaperone to HSP60, normally forming a complex together with unfolded and misfolded substrate proteins, and assists their folding into their native conformation. This ATP-driven process utilizes the hydrophobicity of the inner part of HSP60 that attracts unfolded or misfolded proteins. Earlier studies have however suggested that HSP10 participates in initial unfolding of client proteins and deliver these to HSP60 (Moparthi et al., 2014; Moparthi et al., 2016). HSP10 in humans is normally a mitochondrial protein but has also been found circulating in the blood stream. The presence in the blood arises, 24 h after gestation, thereby giving it the name early pregnancy factor (EPF) (Cavanagh and Morton, 1994). HSP10 is highly abundant in many tissues and is in particular more abundant in the brain than HSP60 (Figure 2). Elevated levels of HSP10 have been found in the NDs with Alzheimer’s disease and Parkinson’s disease. In Alzheimer’s disease, the protein levels of HSP10 rise significantly, while HSP60 stays constant (Hashimoto et al., 2012). In Parkinson’s disease, HSP10 has been found to be entrapped by α-synuclein fibrils (Szegõ et al., 2019). The abundant presence of HSP10 without HSP60 is intriguing and suggests that the complete function of HSP10 is not fully appreciated. Our results indicate that HSP10 has a chaperone function on its own, not merely functioning as a co-chaperone for HSP60. This hypothesis is supported by previous studies of folding and unfolding activity on five different substrate proteins by GroES (carbonic anhydrase, lysozyme, serum albumin, and the bacterial actin homolog MreB) (Moparthi et al., 2014; Moparthi et al., 2016). Furthermore, the mere abundance of HSP10 in relation to HSP60 in tissue throughout the human body (Figure 2) is highly suggestive of HSP10 with separate functions. We proposed that HSP10 is a patrolling chaperone where it functions as an aggregation modulator leading to a suppression of fibrillation when high concentration of HSP10 is present. Since the fibril chaperoning activity of HSP10 can be seen for both Aβ1–42 and PrP the chaperoning mechanism is possibly a general mechanism targeting fibrillar conformers. The three HSP10 homologs also lead to distinct effects in aggregation inhibition. In all ThT fibrillation assays, the most pronounced inhibitory effect was observed for DrHSP10 followed by HuHSP10, while GroES only had a measurable inhibitory effect during PrP aggregation, while also binding to Aβ1–42 fibrils. This difference in efficacy was consistent in the immobilized fibril binding assay, where DrHSP10 shows the best binding to Aβ1–42 fibrils followed by HuHSP10 and then GroES. The low B-values suggest binding targeting fibril ends. The kinetic analysis of DrHSP10 and HuHSP10 during Aβ1–42 fibrillation suggests that the chaperones primarily impact primary nucleation and elongation; however, it is worth noting that these are combined rate constants. That would indicate three possible scenarios: (1) primary nucleation and elongation are both inhibited or (2,3) either primary nucleation or elongation is inhibited. Influence on elongation is compatible with fibril end binding. Influence on nucleation is also compatible with this mechanism considering fibril end stabilization of a fibrillar seed with one open end for growth (Figure 6F). Our data are consistent with expected chaperone activity because chaperones need rather rapid on-and-off rates with rather weak affinities in their function as folding enzymes. Why DrHSP10 is a more efficient fibril inhibiting chaperone than HuHSP10, which is more efficient than GroES is currently not known but is likely associated with a combination of mobile-loop sequence and protein dynamics within the heptameric structure. Furthermore, fibrils formed in the presence of HSP10s displayed altered morphology. Morphological alterations by GroEL modulating α-synuclein fibrils have been reported previously (Fukui et al., 2016). Notably, α-synuclein contains the sequence 67–71 GGAVV within the NAC region of the protein. For Aβ1–42 fibrils formed in the presence of DrHSP10, the morphological change can be seen by fluorescence microscopy as distinct structures compared to all other fibrils assayed in this study. LCO staining confirmed this morphological observation, and DrHSP10 appeared to promote significantly higher qFTAA binding compared to all other Aβ1–42 fibrils. Aβ1–42 fibrils formed in the presence of HuHSP10 may indicate slightly increased qFTAA binding. Nonetheless, all HSP10s promoted formation of bundled fibrils according to TEM. The presence of DrHSP10 and GroES in addition led to amorphous aggregation of Aβ1–42 proposing interference of non-fibrillar species a feature, which would likely be invisible to amyloid ligands.

Of particular interest from the structural hypothesis of avid binding of heptameric HSP10 mobile loops targeting short fibril sequences in Aβ1–42 and PrP, we speculated that this explains how low concentrations of HSP10 chaperones accelerated fibrillation initiation. Essentially, the chaperones under such conditions could work as seed stabilizers templating the primary nucleation for fibril formation, i.e., stabilizing the transient rate-determining step. At higher concentrations of chaperone, this affinity for fibril ends, as expected, inhibits fibril nucleation and growth. The shift between fibril acceleration and deceleration of DrHSP10 and HuHSP10 appears between 10 and 100 nM chaperone, i.e., at a chaperone ratio of 1:500 and 1:50 compared with Aβ1–42. Hence, one can envision that regulating proteostasis by chaperone titration is certainly a balancing act during stress and aging (Figure 10) (Tittelmeier et al., 2020). A prediction from our data is that HSP10 chaperone depletion could accelerate fibril nucleation leading to exacerbated proteostasis impairment. Furthermore, the rapid upregulation of HSP10 during gestation in females implicates a possible druggable path for treatment. More studies on this topic would be very interesting for studies of ND.

## Data Availability Statement

The raw data supporting the conclusions of this article will be made available by the authors, without undue reservation.

## Author Contributions

JL conceived the study, performed most experiments, analyzed data, and wrote the first draft of the manuscript. SN performed experiments and edited the manuscript and figures. PH conceived the study, designed experiments, and wrote the final manuscript. All authors contributed to the article and approved the submitted version.

## Conflict of Interest

The authors declare that the research was conducted in the absence of any commercial or financial relationships that could be construed as a potential conflict of interest.

## Publisher’s Note

All claims expressed in this article are solely those of the authors and do not necessarily represent those of their affiliated organizations, or those of the publisher, the editors and the reviewers. Any product that may be evaluated in this article, or claim that may be made by its manufacturer, is not guaranteed or endorsed by the publisher.
