# Supplementary material for: HSP10 as a Chaperone for Neurodegenerative Amyloid Fibrils
Source: Front Neurosci. 2022 Jun 13;16:902600. doi: 10.3389/fnins.2022.902600 (PMC9234269; doi:10.3389/fnins.2022.902600)
Supplement: Supplementary file 1 [file Data_Sheet_1.PDF]

Supplementary Material

1 Supplementary Data

Contains three supplementary Figures:

SFig.1. shows MS spectra of the different HSP10 chaperones A) GroES, B) HuHSP10 and C) DrHSP10. D) Table, comparing the theoretical mass of the chaperones with the observed mass. Peaks from stable dimers and trimers can also be seen in the spectra. DrHSP10 has a truncation, leading to two monomeric peeks. Yet this does not seem to affect the ability of the chaperone to form its heptameric complex, since stable dimers and trimers can be seen in the spectra. These dimers and trimers are formed both in the absence and presence of the truncated chaperone. HuHSP10 and DrHSP10 are expected to have their starting methionine removed, since the subsequent amino acid in the sequence is an alanine.

SFig. 2 shows an alignment of the sequences GroES, HuHSP10 and DrHSP10. GroES and DrHSP10 were both aligned towards HuHSP10. All the sequenced contained a TEV-protease cleavage site and a 6-His-tag. The sequence alignment was done in Benchling.

SFig. 3 shows all the fibril images used when conducting the hyperspectral micrographs analysis. Fibrillation was conducted in the absence and presence of different chaperones and stained with qFTAA and hFTAA.

SFig 1

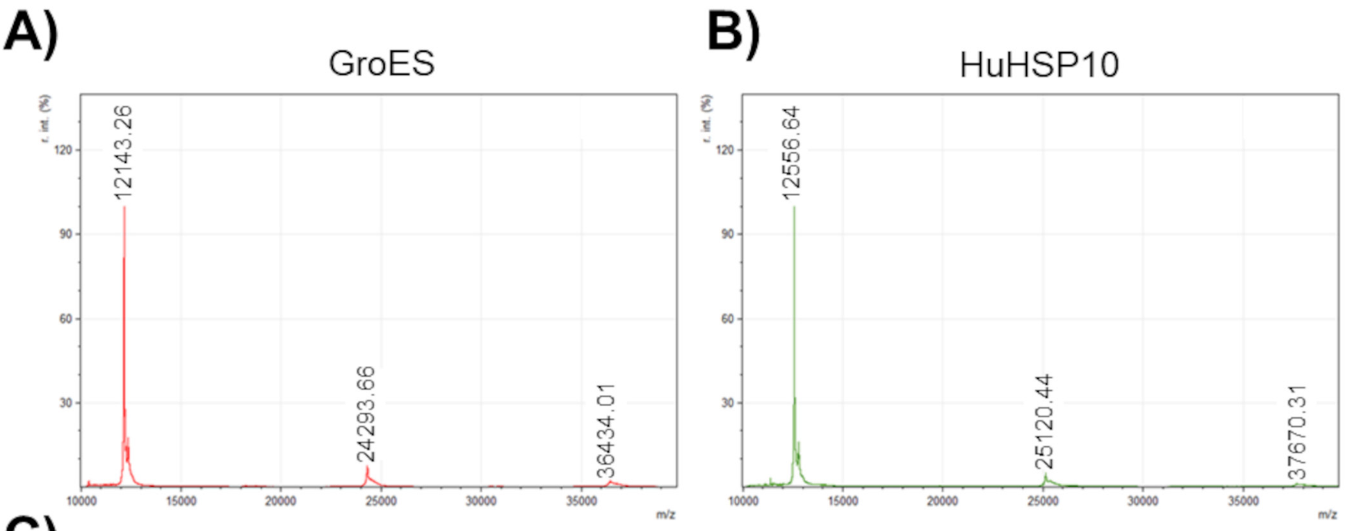

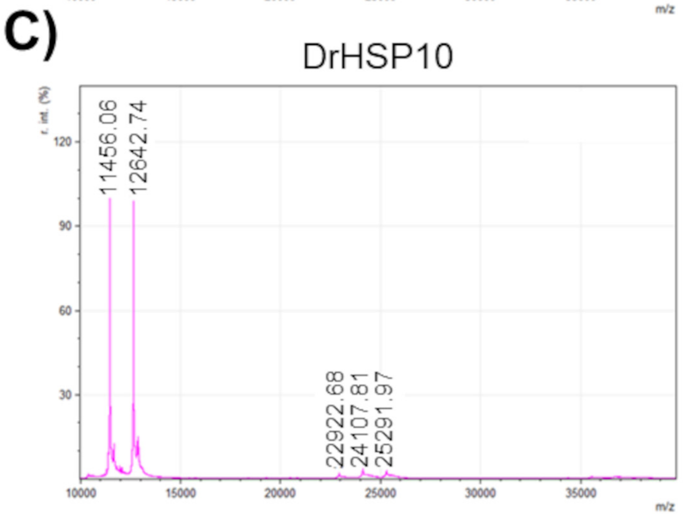

D)

	Theoretical mass	Observed mass
GroES	12148.7	12143.26
HuHSP10 -Met	12562.3	12556.64
DrHSP10 -Met	12646.6	11456.06/ 12642.74

SFig.1. MS spectra of the different chaperones **A)** GroES, **B)** HuHSP10 and **C)** DrHSP10. **D)** Table comparing the theoretical mass of the chaperones with the observed mass. Peaks from stable dimers and trimers can also be seen in the spectra. DrHSP10 has a truncation, leading to two monomeric peeks. Yet this does not seem to affect the ability of the chaperone to form its heptameric complex, since stable dimers and trimers can be seen in the spectra. These dimers and trimers are formed both in the absence and presence of the truncated chaperone. HuHSP10 and DrHSP10 are expected to have their starting methionine removed, since the subsequent amino acid in the sequence is an alanine.

SFig 2

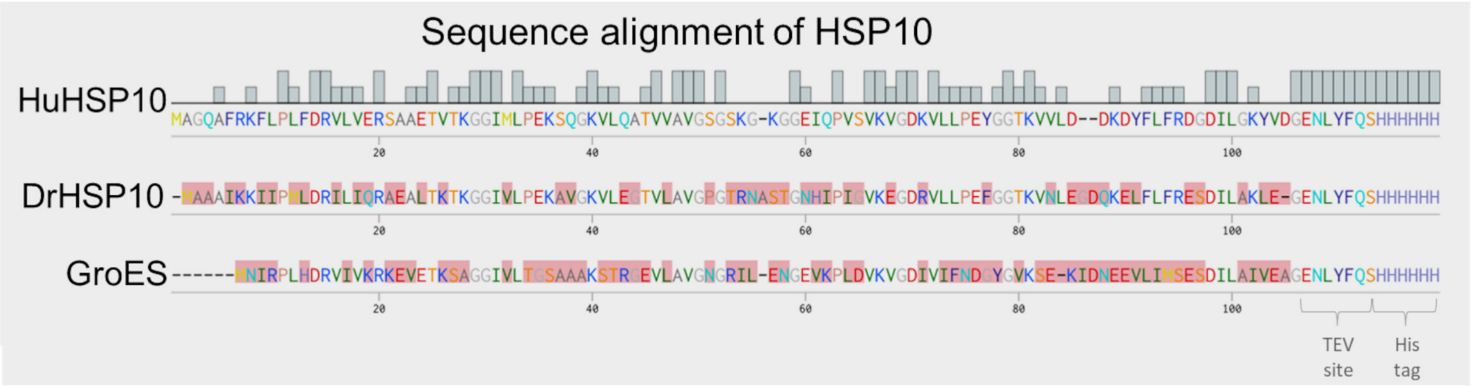

SFig. 2. Sequence alignment of GroES, HuHSP10 and DrHSP10. GroES and DrHSP10 were both aligned towards HuHSP10. All the sequenced contained a TEV-protease cleavage site and a 6-His-tag. The sequence alignment was done in Benchling.

SFig 3

5 μM Aβ1-42 fibrils stained with qFTAA hFTAA in absence and precence of 1 μM chaperone

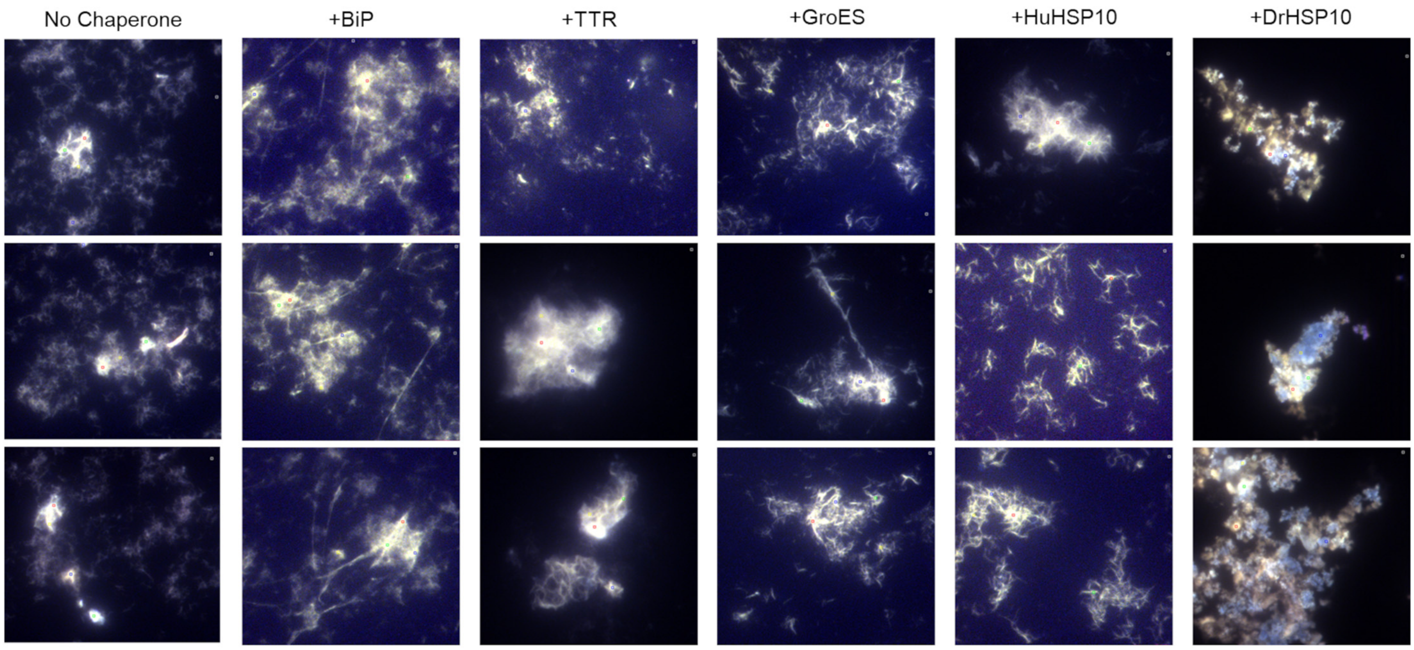

SFig. 3. All hyperspectral images of fibrils for the analysis of ROIs (500/540 nm) qFTAA and hFTAA spectra. Fibrillation was conducted in the absence and presence of different chaperones and thereafter stained with qFTAA and hFTAA.
